# Retraction: Dispersed Ag_2_O/Ag on CNT-graphene composite: an implication for magnificent photoreduction and energy storage applications

**DOI:** 10.3389/fchem.2025.1729486

**Published:** 2025-10-29

**Authors:** Frontiers Editorial Office

The journal retracts the 03 July 2018 article cited above.

Following publication, concerns were raised regarding fundamental errors present in the methodology of this manuscript. Additionally, concerns have been raised regarding multiple areas of overlap with other papers published by the same authors’ group. The article does not meet the standards of editorial and scientific rigor for *Frontiers in Chemistry*; therefore, the article has been retracted.

This retraction was approved by Chief Editors of *Frontiers in Chemistry* and the Chief Executive Editor of Frontiers. The authors did not agree with this retraction. Frontiers would like to thank the concerned readers who contacted us regarding the published article.

